# Tumour-associated myeloid cells expressing IL-10R2/IL-22R1 as a potential biomarker for diagnosis and recurrence of pancreatic ductal adenocarcinoma

**DOI:** 10.1038/s41416-024-02676-w

**Published:** 2024-04-20

**Authors:** Hyung Keun Lee, So Young Kim, Soo-Hyun Chung, Bongkun Choi, Ji-Eun Kim, Dohee Yoon, Sung Ill Jang, Areum Yeo, Hyun Goo Kang, Jusung Lee, Yoon Ha Choi, Joon Seong Park, Yoolim Sung, Jong Kyoung Kim, Eun-Ju Chang, Dong Ki Lee

**Affiliations:** 1 Severance Institute for Vascular and Metabolic Research, Yonsei University College of Medicine, Seoul, Korea; 2 Institute of Vision Research, Department of Ophthalmology, Yonsei University College of Medicine, Seoul, Korea; 3 College of Pharmacy, Yonsei University, Incheon, Korea; 4 Institute of Biomedical Research, Yonsei University College of Medicine, Seoul, Korea; 5 Department of Biochemistry and Molecular Biology, Brain Korea 21 Project, Asan Medical Center, University of Ulsan College of Medicine, Seoul, Korea; 6 Stem Cell Immunomodulation Research Center, Asan Medical Center, University of Ulsan College of Medicine, Seoul, Korea; 7 Department of Internal Medicine, Gangnam Severance Hospital, Yonsei University College of Medicine, Seoul, Korea; 8 Department of Life Sciences, Pohang University of Science and Technology (POSTECH), Pohang, Korea; 9 Department of New Biology, DGIST, Daegu, Korea; 10 Department of Surgery, Gangnam Severance Hospital, Yonsei University College of Medicine, Seoul, Korea; 11 Department of Surgery and Cancer Research Institute, Seoul National University College of Medicine, Seoul, Korea; 12 Institute for Convergence Research and Education in Advanced Technology, Yonsei University, Seoul, Korea

**Keywords:** Diagnostic markers, Immunosurveillance

## Abstract

**Background:**

Pancreatic ductal adenocarcinoma (PDAC) is an aggressive malignancy with a poor survival rate, largely due to the lack of early diagnosis. Although myeloid cells are crucial in the tumour microenvironment, whether their specific subset can be a biomarker of PDAC progression is unclear.

**Methods:**

We analysed IL-22 receptor expression in PDAC and peripheral blood. Additionally, we analysed gene expression profiles of IL-10R2^+^/IL-22R1^+^ myeloid cells and the presence of these cells using single-cell RNA sequencing and murine orthotropic PDAC models, respectively, followed by examining the immunosuppressive function of IL-10R2^+^/IL-22R1^+^ myeloid cells. Finally, the correlation between IL-10R2 expression and PDAC progression was evaluated.

**Results:**

IL-10R2^+^/IL-22R1^+^ myeloid cells were present in PDAC and peripheral blood. Blood IL-10R2^+^ myeloid cells displayed a gene expression signature associated with tumour-educated circulating monocytes. IL-10R2^+^/IL-22R1^+^ myeloid cells from human myeloid cell culture inhibited T cell proliferation. By mouse models for PDAC, we found a positive correlation between pancreatic tumour growth and increased blood IL-10R2^+^/IL-22R1^+^ myeloid cells. IL-10R2^+^/IL-22R1^+^ myeloid cells from an early phase of the PDAC model suppressed T cell proliferation and cytotoxicity. IL-10R2^+^ myeloid cells indicated tumour recurrence 130 days sooner than CA19-9 in post-pancreatectomy patients.

**Conclusions:**

IL-10R2^+^/IL-22R1^+^ myeloid cells in the peripheral blood might be an early marker of PDAC prognosis.

## Background

Pancreatic ductal adenocarcinoma (PDAC) accounts for 90% of all pancreatic malignancies. PDAC frequency is increasing worldwide, and PDAC is predicted to become the second leading cause of cancer mortality by 2030 [1, 2]. High PDAC mortality is mainly caused by poor early detection and limited late-stage treatment [1, 2]. Moreover, the tumour microenvironment (TME) of PDAC is nonimmunogenic [3, 4], contributing to therapy resistance. Immunosuppressive TME protects PDAC from traditional treatment (e.g. chemotherapy) and immunotherapy that has been successful in other cancers [5]. Hence, identifying specific cell subtypes infiltrating the PDAC TME, associated with PDAC progression, is critical for early detection and more effective therapeutic options to improve the survival of affected patients.

PDAC cells that escape host immune surveillance result a TME promoting tumorigenesis, cancer progression and metastasis. In the late stage, PDAC cells recruit various other cells, such as myeloid cells, including tumour-associated macrophages (TAMs), tumour-associated neutrophils, myeloid-derived suppressor cells and regulatory T cells (Tregs), establishing an immunosuppressive TME [3–5]. In PDAC TME, myeloid cells infiltrating the tumour are predominant and represent crucial mediators of immune evasion, which is associated with poor outcomes [6]. These cells induce T cell dysfunction and recruit Tregs, promoting tumour growth through immunosuppression [7–9]. This immunosuppressive TME impairs the efficacy of immunotherapy. Additionally, cells within the TME produce cytokines that regulate TME and cancer pathogenesis by promoting the infiltration of tumour-associated immune cells [10]. Thus, whether the association between PDAC-infiltrated specific myeloid cells and PDAC progression could be used to establish an early diagnosis of PDAC is important. In our study, we found that IL-10R2^+^/IL-22R1^+^ myeloid cells in human peripheral blood mononuclear cell (PBMC) culture and murine PDAC model inhibited T cell proliferation. Therefore, these cells could be an early diagnostic marker reflecting the immunosuppressive nature of PDAC TME.

Current evidence shows that interleukin-22 (IL-22) is highly expressed in PDAC [11, 12]. IL-22 secreted by T helper 17, innate lymphoid and myeloid cells [13, 14] plays a role in the progression of colon [15], lung [13, 16] and breast cancer [17] and lymphomas [18]. Furthermore, IL-22 promotes pancreatic fibrosis [19] and PDAC development [12, 20]. IL-22 receptor is also elevated in gastric cancer [21], hepatocellular carcinoma [22] and PDAC [11]. However, the clinical benefit of detecting elevated IL-22 and its receptor to diagnose PDAC early or indicate PDAC recurrence remains unknown.

In this study, we demonstrated that IL-22 receptor (IL-10R2/IL-22R1)–expressing myeloid cells are abundant in PDAC patient blood. IL-10R2^+^/IL-22R1^+^ cells in the blood expressed tumour-educated monocyte (TEM) signature genes and infiltrated PDAC. Additionally, IL-10R2^+^/IL-22R1^+^ cells in human myeloid cell culture inhibited T cell proliferation in vitro. In murine PDAC models, early pancreatic tumour progression was correlated with IL-10R2^+^/IL-22R1^+^ cell infiltration of PDAC. Furthermore, IL-10R2^+^/IL-22R1^+^ cells from the early stage of the PDAC model inhibited T cell proliferation and cytotoxicity. Finally, IL-10R2^+^/IL-22R1^+^ myeloid cells in the blood could be a reliable indicator for PDAC prognosis and a predictive marker of PDAC recurrence.

## Methods

### Patient inclusion and sample collection

We collected peripheral blood from patients with PDAC, acute (AP) and chronic pancreatitis (CP), intraductal papillary mucinous neoplasm (IPMN) and other malignancies. PDAC and other cancers were diagnosed by cytology using endoscopic ultrasound-guided fine needle aspiration (EUS-FNA) or surgical specimens. AP, CP, and IPMN were diagnosed by evaluating clinical symptoms and imaging, including computed tomography (CT), EUS and/or magnetic resonance imaging (MRI).

Initial blood samples were obtained at diagnosis in all subjects, including PDAC (*n* = 154), AP (*n* = 32) or CP (*n* = 39), bile duct cancer (*n* = 57) and IPMN (*n* = 9) cases. Non-cancer participants (*n* = 159) provided control samples. In patients with resectable PDAC, blood samples were collected monthly after surgery for 12 months and at recurrence during follow-up. In unresectable PDAC cases, blood samples were collected every three months during chemotherapy/radiotherapy. Patients with serious illnesses, immunosuppression or autoimmune/infectious diseases were excluded. Approximately 10 mL of blood was collected in ethylenediaminetetraacetic acid tubes for each subject.

The following clinical characteristics of PDAC patients were collected: sex, age, smoking status, alcohol consumption, history of diabetes, carcinoembryonic antigen (CA19-9) level at diagnosis, primary location of pancreatic cancer, resectability at diagnosis, initial treatment modality and tumour stage (Table S1 in Additional file 1). The clinical tumour stage was determined according to the TNM classification system for PDAC [23]. An appropriate committee approved this study as described in the ***Ethics approval and consent to participate***, and all participants provided informed consent.

### Study design

The robustness of the study was fortified through careful consideration of sample size determination, randomisation and blinding procedures.

#### Sample size determination

The sample size was determined to ensure adequate statistical power for detecting a pre-specified effect size. This involved estimating the effect size based on preliminary data. Significance level (α) and desired power (1 - β) were pre-specified, and corresponding type I and II error rates were determined. The sample size calculation also included the estimation of variability within the data. The entire process was pre-specified in the study protocol to maintain transparency and reduce the risk of bias. While formal statistical methods were not employed for animal studies, the inherent variability in animal responses, ethical concerns and practical constraints were considered during sample size estimation. We aimed at a balance between obtaining meaningful results and minimising the number of animals used. This process aligns with ethical standards and good research practices, contributing to the reliability and ethical conduct of our animal studies.

#### Randomisation

Randomisation, particularly simple randomisation, was used to allocate samples or animals to different experimental groups. Allocation concealment was implemented to prevent bias, monitoring the process to ensure adherence to the randomised assignment. In animal studies, while formal randomisation was not employed, we acknowledge its absence and the use of practical considerations for allocation. This recognition is essential for transparency in our study design.

#### Blinding

Investigators were blinded to group allocation throughout the study to enhance its objectivity, aiming to mitigate potential bias to ensure that those conducting the experiment and evaluating outcomes remained unaware of group assignments. While no formal blinding was implemented in animal studies, we acknowledge its absence. Furthermore, group assignments and outcome assessments were not blinded. This recognition is crucial for transparency in our study design and interpretation of results.

### Reagents and antibodies

Table S2, in Additional file 2, describes the reagents and antibodies used in this study.

### Flow cytometry

Human PBMCs were isolated from whole blood using Histopaque® 1077 or Histopaque® 1083, both with 1119 density gradient centrifugation. The interphase layers of PBMCs and granulocytes were washed in phosphate-buffered saline containing 0.5% bovine serum albumin. Mouse peripheral blood cells were prepared by treating blood cells with red blood cell lysis buffer two times. Tumour tissues were treated with collagenase and dispase (human) or collagenase and DNase I (mouse) to prepare cell suspensions. The cells were stained with Fc blockers and antibodies and analysed with a FACS Canto II cytometer. Data were analysed using FlowJo (Ver10).

### mRNA collection and quantitative RT-PCR

Total RNA was extracted from human PBMCs using QIAzol reagent and reverse transcribed to cDNA using a cDNA Synthesis Kit. Quantitative real-time PCR was performed using a PCR detection system (StepOnePlus Real-Time PCR) and a commercial detection kit (SYBR Premix Ex Taq Kit). Table S3 in Additional File 3 provides the primer sequences used in this study.

### FACS cell sorting

Single-cell suspensions of human PBMCs, PBMC culture, or mouse spleens from Pan02-inoculated mice 3 days before the sorting, were stained with fluorochrome-conjugated antibodies and appropriate isotype controls. Afterwards, the cells were sorted based on fluorescence intensity using a BD FACSAria III cell sorter.

### scRNA-seq experiments

IL-10R2^+^ cells were enriched from PBMCs of PDAC patients using a FACSAria III flow cytometer. Sorted cells were stained with trypan blue and diluted at 1×10^5^–2×10^6^ cells/mL for cell counting and viability check. Cell viability was approximately 90%. Single-cell RNA sequencing (scRNA-seq) libraries were generated using the Chromium system (10x Genomics) with the Chromium Single Cell 3’ Library & Gel Bead Kit v2.

### scRNA-seq data analysis

Raw reads were aligned to the GRCh38 human reference genome (Ensembl release 91) by the Cell Ranger software (v2.2.0) using default parameters. Cell barcodes corresponding to empty droplets were filtered using the emptyDrops function of the DropletUtils (v1.2.2) R package with a false discovery rate <0.01. Low-quality cells with >10% unique molecular identifiers mapped to mitochondrial genes, <1,000 total unique molecular identifier counts and <102.5 expressed genes were excluded. The thresholds were chosen by visually inspecting outliers in the two-dimensional principal component analysis plot on all quality control metrics calculated by the perCellQCMetrics function of the scatter (v1.10.1) R package. The filtered count matrix was normalised using the Seurat (v3.0-alpha) R package. In each sample, the top 2,000 highly variable genes were selected as feature genes using the FindVariableFeatures function of the Seurat package with default options. Batch effects were removed using the Seurat package on 30 canonical correlation vectors. The integrated expression matrix was scaled and then visualised in the two-dimensional UMAP plot using the RunUMAP function of the Seurat package on 30 principal components. For cell type annotation, the CreateSingleRSeuratObject function of the SingleR R package (v.0.2.2) [24] was performed with the following parameters: npca=15, min.cells=0, min.genes=0 and regress.out=NULL. Differentially expressed genes between P5 and P5(−) samples or cell type–specific marker genes were identified using the FindAllMarkers function of the Seurat package with an adjusted *P* value < 0.01. The raw unique molecular identifier count matrices of PBMCs from controls (2.1.0/pbmc8k, 1.1.0/pbmc6k, 1.1.0/frozen_pbmc_donor_a, 1.1.0/frozen_pbmc_donor_b, 1.1.0/frozen_pbmc_donor_c) were downloaded from 10X Genomics datasets (https://www.10xgenomics.com/resources/datasets).

### Laboratory animals

Male C57BL/6 mice (6–7-week-old) were obtained from the Jackson Laboratory. The animals were maintained in a specific pathogen-free environment, acclimated for at least one week before any experiments and randomly assigned to treatment groups. All animal experiments were approved by the appropriate committee as described in the ***Ethics approval and consent to participate***.

### Cell lines and culture

Murine pancreatic cancer cell line (Pan02 cells) was purchased from ATCC and cultured in RPMI 1640 (containing 20 mM HEPES and L-glutamine, without sodium bicarbonate) supplemented with 10% heat-inactivated foetal bovine serum and 1% penicillin-streptomycin at 37 °C in a humidified atmosphere of 5% CO_2_. We cultured human PBMC cells with GM-CSF and IL-6 (20 ng/mL) for four days to prepare human myeloid cell–derived suppressor cell (MDSC) culture as described elsewhere [25, 26]. As a functional control, we cultured human PBMC cells with GM-CSF (20 ng/mL) only (inflammatory monocytes).

### Animal model for cancer induction (murine orthotopic model)

Mice were separately placed in an anaesthetising chamber and anaesthetised with 2%–3% isoflurane in 100% oxygen. We orthotopically implanted 1×10^6^ Pan02 cells into the mouse pancreas. The mice were sacrificed on a scheduled date.

### Immune suppression assay

Immune suppression assay was performed according to a protocol adapted from previous reports [25, 26]. For human cells, T cells were prepared by culturing PBMC cells with IL-2 (10 ng/mL) for 4 days, labelling them with CFSE (2 μM) and coculturing with myeloid cells at a 1:20 ratio in a 96-well round bottom plate. After 3 days, the cells were harvested and stained with anti-CD8 antibody. The CFSE level was analysed by flow cytometry. For murine cells, a 96-well round bottom plate was coated with 2 μg/mL of anti-CD3 antibody at 4 °C overnight. Lymph nodes and the spleen were harvested from C57BL/6 mice, and T cells were enriched by a mouse T cell enrichment kit. The cells were further labelled with CFSE (2 μM). Sorted murine myeloid cells were cocultured with CFSE-labelled T cells at a 1:20 ratio and anti-CD28 antibody (2 μg/mL). After 3 days, the cells were harvested, and CFSE and granzyme B expression was analysed by flow cytometry.

### Immunohistochemical (IHC) staining

The IHC staining for histologic sections (5 μm) was previously described [27]. Trichrome and Picro Sirius Red staining was performed following the standard protocol provided by the manufacturer.

### Statistical analysis

The Chi-square test was used to compare frequencies. Continuous variables were examined for normality using a Shapiro-Wilk test. Normal variables were presented as a mean and standard deviation (SD) and compared using an independent two-sample *t*-test, paired *t*-test or one-way analysis of variance. The assumption of equal variances for continuous variables subjected to one-way ANOVA was checked using Levene’s test. Confirming homogeneity of variances justified the use of ANOVA for group comparisons. Nonnormal variables were presented as a median and interquartile range and assessed using the Mann-Whitney U, Wilcoxon signed rank or Kruskal-Wallis test. Post-hoc p-values were corrected with the Bonferroni method. Correlation coefficients between two continuous variables were calculated by Pearson correlation analysis.

The independent predictors of PDAC were determined using multivariable logistic regression: variables showing *p*-values < 0.05 by univariable logistic regression were entered. The area under the curve (AUC) for a receiver operating characteristic curve determined the predictive ability of IL-10R2, IL-22R1 and IL-22. A combination of IL-10R2, IL-22R1 and IL-22 for PDAC was compared using the DeLong method. Kaplan-Meier survival curves were created using overall survival data. The groups were compared using the log-rank test. Two-sided p-values < 0.05 were considered significantly different. All statistical analyses were performed using SAS (version 9.4, SAS Inc., Cary, NC) and SPSS for Windows, version 25.0 (SPSS, Chicago, IL).

## Results

### Concomitant elevation of IL-22 in PDAC and IL-10R2^+^/IL-22R1^+^ cells in blood samples

**Fig. 1:**
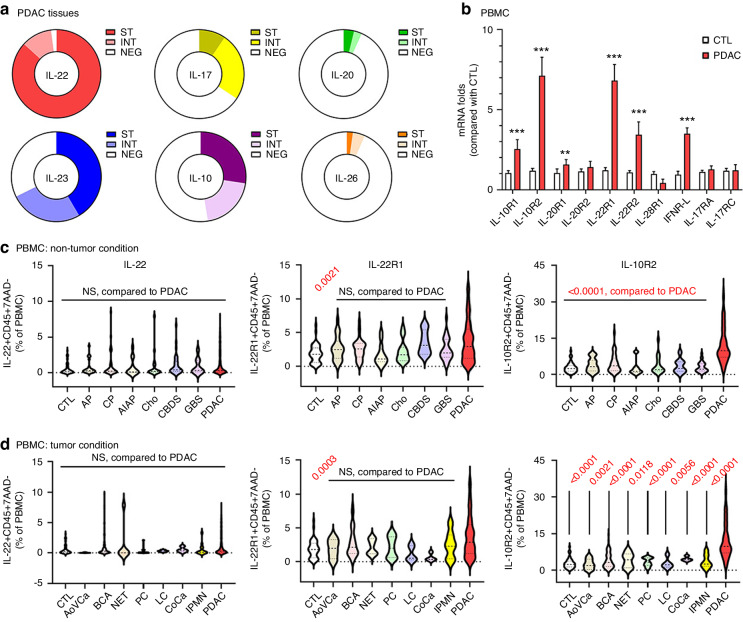
Concomitant elevation in IL-22 in PDAC and IL-10R2^+^/IL-22R1^+^ cells in PDAC patient peripheral blood. **a** Pie charts represent the fractions of IL-10 family cytokines (ST: strong, INT: intermittent, NEG: negative staining), as evaluated by TMAs from PDAC patients (*n* = 184). **b** Gene expression levels of receptors that bind to IL-10 cytokines were analysed by qPCR from naïve (CTL) and PDAC PBMCs. qPCR experiments were repeated three times with triplicate samples (mean ± SD, **p < 0.01; ***p < 0.001; Student *t*-test). (**c**, **d**) Comparison of the expression of IL-22^+^, IL-10R2^+^ or IL-22R1^+^CD45^+^ cells in PBMCs under nontumorous (**c**) or tumorous conditions of the pancreas (**d**) by flow cytometry (one-way ANOVA with Tukey’s post hoc multiple comparison tests; each p-value for each disease were compared with PDAC; NS: not significant). CTL; control (*n* = 98), AP; acute pancreatitis (*n* = 25), CP; chronic pancreatitis (*n* = 36), AIAP; alcohol-induced acute pancreatitis (n = 13), Cho; cholangitis (*n* = 52), CBDS; common bile duct stone (*n* = 31), GBS; gall bladder stone (*n* = 42), PDAC; pancreatic ductal adenocarcinoma (*n* = 180), AoVCa; ampulla of Vater carcinoma (*n* = 5), BCA; biliary carcinoma (*n* = 91), NET; neuroendocrine tumour of the pancreas (*n* = 6), PC; pancreatic cyst or pseudocysts (*n* = 23), LC; lung cancer (*n* = 15), CoCa; colon cancer (*N* = 15), IPMN; intraductal papillary mucinous neoplasm (*N* = 13).

We determined IL-10 family cytokine expression by IHC using PDAC tissue microarray (TMA) blocks to evaluate the association between the expression of tumour-driven immunosuppressive cytokines and PDAC. Cancer tissues from 183 patients (99.5%) exhibited strong staining for at least one of these cytokines (Fig. 1, Figure S1 in Additional file 4). IL-22 was the most frequently expressed cytokine (96.8% of patients, Fig. 1a and Figure S1) and showed strong staining in tumour and peritumour matrix tissues (strong expression [ST]: 82.3%) (Fig. 1a and Figure S1). In contrast, positive staining for IL-10, IL-17 and IL-23 was observed in 47.2%, 29.3% and 69.4% of patients, respectively. Moreover, staining of <10% was found for IL-20 and IL-26 in PDAC patients (ST: 9.3% and 2.9%, respectively) (Fig. 1a).

IL-22 signals through a heterodimeric receptor composed of two subunits, IL-10R2 and IL-22R1 [28]. Given the significant elevation of IL-22 in PDAC, we determined whether the population of IL-10R2^+^/IL-22R1^+^ cells was increased in PDAC patient PBMC samples by detecting the mRNA expression of IL-10 family receptors compared to controls. We observed an approximately 7-fold increase in IL-10R2 and IL-22R1 mRNA levels in PBMCs of PDAC patients compared to controls (Fig. 1b). IL-10R1, IL-20R1, IL-22R2 and IFNR-λ mRNA levels were significantly elevated in PDAC patients (Fig. 1b). These results show that mRNA expression of IL-22 receptors, including IL-10R2 and IL-22R1, is highly elevated in PBMCs of PDAC patients compared to controls.

Given the elevated mRNA expression of IL-10R2 and IL-22R1 in PDAC PBMCs, the surface protein expression of these receptors in PDAC PBMCs might be higher than those from controls and patients with other disorders. Using flow cytometry, the levels of IL-22^+^, IL-10R2^+^ or IL-22R1^+^ cells in PBMCs from PDAC patients were compared to controls and patients with other disorders, including AP, CP, alcohol-induced acute pancreatitis (AIAP), cholangitis (Cho), common bile duct stone (CBDS) and gall bladder stone (GBS). IL-10R2^+^ cells expressing CD45 (IL-10R2^+^CD45^+^ cells) were specifically elevated in PDAC patients compared to all other patients (Fig. 1c). When the three markers were compared to other malignancies, including ampulla of Vater carcinoma (AoVCa), biliary carcinoma (BCA), neuroendocrine tumour of the pancreas (NET), pancreatic cyst or pseudocysts (PC), lung cancer (LC), colon cancer (CoCa) and IPMN, only IL-10R2^+^ cells were significantly elevated in PDAC (Fig. 1d). In contrast, the number of IL-22R1^+^ cells was not significantly different between PDAC and other tested diseases. However, IL-22R1^+^ cells were significantly elevated in PDAC patients compared to controls (*p* = 0.0021 and 0.0003, Fig. 1c, d). Therefore, a correlation exists between elevated IL-22 expression in PDAC and an abundant population of IL-10R2^+^ or IL-22R1^+^ cells in the peripheral blood of PDAC patients. Moreover, IL-10R2^+^ cells were identified as a specific marker distinguishing PDAC from other malignancies.

### Characterisation of IL-10R2^+^ myeloid cells in the peripheral blood of human PDAC patients by scRNA-seq and their infiltration of PDAC

**Fig. 2:**
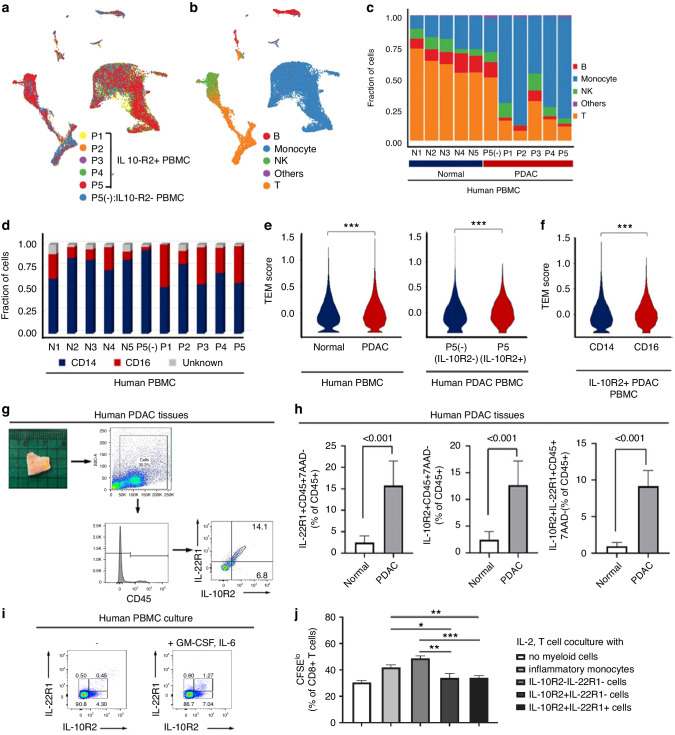
Characterisation of IL-10R2^+^ cells in PDAC patients’ blood by scRNA-seq, and their infiltration in PDAC and an immunosuppressive function. **a** UMAP plots of 24,819 IL-10R2^+^ PBMCs for five patients (P1–5) with PDAC and of 3,721 IL-10R2^-^ PBMCs for one patient with PDAC as a control (P5[−]). **b** UMAP plots of immune cell type. **c** Immune cell-type distribution for 33,981 PBMCs from five healthy donors (N1–5), 3721 IL-10R2^-^ PMBCs from one PDAC patient (P5[−]) and 18,608 IL-10R2^+^ PBMCs from five PDAC patients (P1–5). **d** Cell subtype distribution of PBMCs within the mono/macrophage compartment with CD14 and CD16. **e** Violin plots show TEM signature scores for the monocyte/macrophage compartment in controls and PDAC patients (left), as well as in IL-10R2^−^ (P5[−]) and IL-10R2^+^ (P5) PBMCs from PDAC patients (right) (***p < 10^−8^ using the Wilcoxon rank sum test). **f** Violin plots showing TEM signature scores for the monocyte/macrophage compartment in CD14^+^ and CD16^+^ subtypes of IL-10R2^+^ PBMCs from PDAC patients (***p < 2.2 × 10^−16^ using the Wilcoxon rank sum test). **g** FACS analysis strategies for human PDAC tissue samples. PDAC cells were gated on lymphocytes (CD45^+^), and IL-22R1 and IL^-^10R2 expression was presented. **h** IL-22R1^+^ CD45^+^ (left), IL-10R2^+^CD45^+^ (middle) and IL-22R1^+^IL-10R2^+^ CD45^+^ (right^)^ cell numbers between normal (normal) and PDAC pancreatic tissue (*n* = 6) were determined by flow cytometry (Mann-Whitney U test). **i** IL-22R1, IL-10R2 expression on human PBMCs (left) or MDSC culture (GM-CSF + IL-6, right) were determined by flow cytometry. **j** IL-10R2- and IL-22R1-expressing cells were sorted from the MDSC culture, and T cell proliferation was examined in coculture of myeloid cells and T cells. Means and SDs of triplicate culture were presented. *p < 0.05, **p < 0.01, ***p < 0.001 by students’ *t*-test.

Given IL-10R2^+^ PBMC enrichment in PDAC, we analysed these cells by scRNA-seq to further identify their specific enriched subtype, considering their heterogeneity (Figure S2 in Additional file 4). scRNA-seq was performed using IL-10R2^+^ PBMCs from five PDAC patients (Figure S3a in Additional file 4) and IL-10R2^−^ PBMCs from controls (P5). A total of 24,819 cells (18,608 IL-10R2^+^ and 3,721 IL-10R2^−^ cells) passed our quality control criteria and were represented in a low-dimensional space using the UMAP algorithm [29] (Fig. 2a). We applied the SingleR algorithm [30] to identify four major immune cell types: B, T and natural killer cells, as well as monocytes/macrophages, which were confirmed by the expression of cell type–specific markers (Fig. 2b, Figure S3b, c in Additional file 4). The composition of the IL-10R2^−^ cells in PDAC patients was similar to that of controls (Fig. 2c). Compared to controls and IL-10R2^−^ cells (P5[−]) in PDAC patients, we identified an expansion of monocytes/macrophages and a reduction in B and T cells in IL-10R2^+^ cells of PDAC patients (Fig. 2c).

Given the identified proportion of monocytes/macrophages among IL-10R2^+^ PBMCs, we further divided and characterised this subpopulation. We analysed CD14 and CD16 expression, two surface markers categorising monocytes into classical (CD14^+^CD16^−^), intermediate (CD14^+^CD16^+^) and nonclassical (CD14^−^CD16^+^) types, to determine whether this subset belongs to the myeloid population. Additionally, monocyte/macrophage subsets from IL-10R2^+^ cells were distinguished by CD16 expression, indicating enrichment of nonclassical monocytes compared to IL-10R2^−^ cells (P5[−]) (Fig. 2d and Figure S4a, b in Additional file 4). This subset exhibited high TNFSF10 levels, which belong to a signature gene set associated with the recently characterised TEM [31]. Additionally, *CSF1R*, *CX3CR1*, *CXCL16*, *IL1B*, *IL15*, *IL1R1*, *IL2RG* and *TNFRSF8*, which are involved in further differentiation to TAM [32–34], showed high intensity in this subset (Figure S4c in Additional file 4). The TEM score for IL-10R2^+^ monocytes from PDAC patients was significantly higher than that of all monocytes from controls or IL-10R2^−^ monocytes (*p* < 10^−8^, Fig. 2e), possibly explained by the increased proportion of nonclassical (CD14^−^CD16^+^) monocytes in the IL-10R2^+^ monocyte/macrophage compartment (Fig. 2f and Figure S4d, in Additional file 4). Thus, IL-10R2^+^ PBMCs of PDAC patients are enriched by myeloid-derived nonclassical monocytes expressing the TEM signature.

The enriched population of IL-10R2^+^ myeloid cells displaying the TEM signature in the peripheral blood of PDAC patients suggested that these cells are predisposed to migrate from the peripheral blood to PDAC during cancer progression. To test this possibility, we compared the level of cells expressing IL-22 binding receptors (IL-10R2^+^ and IL-22R1^+^) between controls and PDAC patients using flow cytometry. The total CD45^+^ cell population represented 2.8%–14.3% of all cells (Fig. 2g). Among CD45^+^ cells in PDAC, IL-22R1^+^ (15.9% of CD45^+^) or IL-10R2^+^ (13.4% of CD45^+^) cells were significantly increased compared to controls (2.3% of IL-22R1^+^CD45^+^ and 3.2% of IL-10R2^+^CD45^+^) (Fig. 2h). In addition to cells positive for a single receptor, 8.6% ± 6.4% (range 0.0–12.2%) of cells had both receptors (IL-22R1 and IL-10R2), i.e. double-positive cells (DPCs) (Fig. 2h), demonstrating the increased infiltration of IL-10R2^+^ cells in PDAC tissues.

PDAC is characterised by the infiltration of immunosuppressive myeloid cells, such as TEMs and TAMs [35–39]. Therefore, IL-10R2^+^ myeloid cells might be immunosuppressive. To address this issue, we prepared a human PBMC culture that mimics TME myeloid cells with an immunosuppressive role [25, 26]. IL-10R2/IL-22R1 expression was increased in this culture (Fig. 2I). Furthermore, we enriched and cocultured these populations with T cells. IL-10R2^+^IL-22R1^−^ and IL-10R2^+^IL-22R1^+^ cells inhibited the proliferation of CD8^+^ T cells compared to inflammatory monocytes and IL-10R2^−^IL-22R1^−^ cells (Fig. 2J).

### Enrichment of IL-10R2^+^/IL-22R1^+^ myeloid cells infiltrating the tumour in a murine model of pancreatic cancer

**Fig. 3:**
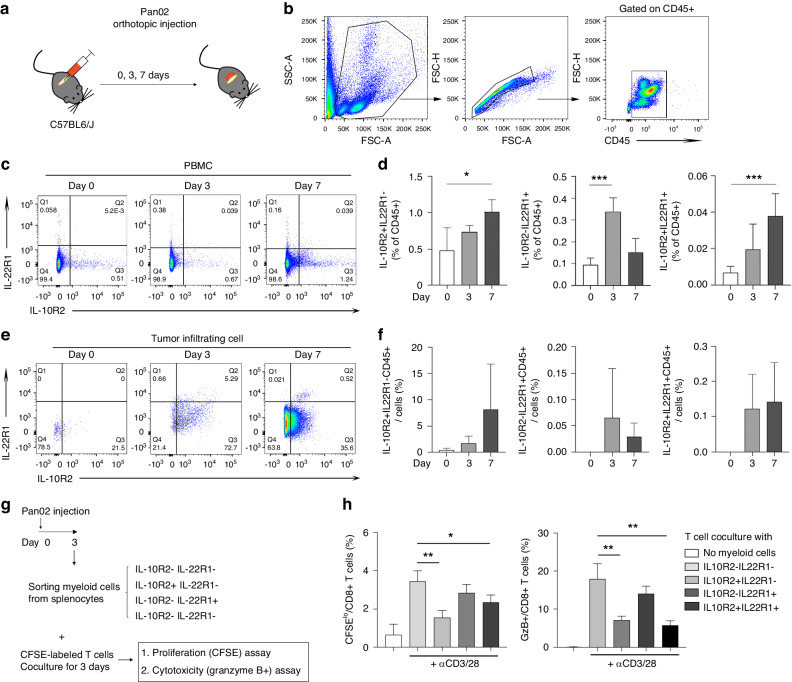
Enrichment of IL-10R2^+^ myeloid cells infiltrating tumour tissues and immunosuppressive functions during pancreatic tumour progression in murine models. **a** A mouse PDAC model was created by orthotopic injection of Pan02 cells into the pancreas of C57BL/6 mice. Four mice each on days 0, 3 and 7 were sacrificed, and blood cells, pancreatic tissue or tumour cells were analysed. **b** Gating strategy for flow cytometry analysis. The cells were gated by front/side scatters and further on singlets (FSC-A and FSC-H), lymphocytes (CD45^+^), followed by analysing IL-10R2 and IL-22R1 expression. **c** Representative figures of IL-10R2 and IL-22R1 expression in CD45^+^ cells from the blood. **d** Abundance of IL-10R2^+^ and IL-22R1^+^ in CD45^+^ cells in the blood. Means and SDs of each mouse at days 0, 3 and 7 were shown. *p < 0.05, ***p < 0.001 by students’ *t*-test. **e** Representative figures of IL-10R2 and IL-22R1 expression in CD45^+^ cells infiltrating the pancreatic tumour (days 3 and 7) or CD45^+^ cells in the pancreatic tissue (day 0). **f** Abundance of IL-10R2^+^CD45^+^ and IL-22R1^+^CD45^+^ cells in tumour tissue. **g** Strategy for the immunosuppression assay for IL-10R2, IL-22R1-expressing myeloid cells. IL-10R2^+/−^IL-22R1^+/−^ myeloid cells were sorted from spleens that were harvested from 4 C57BL/6 mice 3 days after orthotropic injection of Pan02. Each subset was cocultured with CFSE-labelled T cells, followed by measuring proliferation and cytotoxicity of CD8^+^ T cells by flow cytometry. **h** T cell proliferation (CFSE^lo^, left) and cytotoxicity (Granzyme B [GzB]^+^, right). Means and SDs of quadruplicate culture were presented. *p < 0.05, **p < 0.01 by students’ *t*-test.

We established a murine pancreatic cancer model by orthotopically injecting Pan02 cells into C57BL/6 mice to verify whether the population of IL-10R2^+^/IL-22R1^+^ myeloid cells was augmented in a PDAC murine model in a similar manner to human PDAC patients (Fig. 3a) [40, 41]. IL-10R2^+^ or IL-22R1^+^ cells in PBMCs were analysed by flow cytometry (Fig. 3b). IL-10R2^+^IL-22R1^−^CD45^+^ cells were gradually increased from day 3 to day 7; however, IL-10R2^−^IL-22R1^+^CD45^+^ cells peaked at day 3 and decreased at day 7 (Fig. 3c, d). Additionally, both single-positive and IL-10R2^+^IL-22R1^+^ cells were increased in PBMCs on days 3 and 7 (Fig. 3d).

Then, we evaluated tumour-infiltrated IL-10R2^+^CD45^+^ or IL-22R1^+^CD45^+^ cells by flow cytometry (Fig. 3e, f). IL-10R2^+^IL-22R1^−^ cells among CD45^+^ cells continuously increased on days 3 and 7 (Fig. 3e, f), correlating with the enrichment of this cell population in PBMCs (Fig. 3c, d). However, IL-10R2^−^IL-22R1^+^ cells increased on day 3 and decreased on day 7 (Fig. 3e, f), also correlating with those in PBMCs. IL-10R2^+^IL-22R1^+^ DPCs, accounting for a smaller percentage in PBMCs, were more abundant tumour tissues at days 3 and 7 (Fig. 3e, f). Therefore, pancreatic tumour progression in a murine model also correlated with increased IL-10R2^+^/IL-22R1^+^ myeloid cells and their infiltration in tumour tissues, as seen in human PDAC. Furthermore, an increase in the IL-10R2^+^ cell population reflects tumour progression, whereas a momentary surge in IL-22R1^+^ cells is linked to the early detection of PDAC.

### Immunosuppressive roles of IL-10R2^+^/IL-22R1^+^ myeloid cells from tumour-bearing mice

Since IL-10R2^+^ cells from PDAC patients showed a high TEM score and TAM signature gene expression profile (Fig. 2e), which could suppress T cell proliferation (Fig. 2i, j), IL-10R2^+^ and IL-22R1^+^ myeloid cells in PDAC TME might be immunosuppressive. Therefore, we sorted IL-10R2^+^/IL-22R1^+^ myeloid cells from tumour-bearing mice in an early phase of the PDAC orthotopic model and cocultured them with T cells (Fig. 3g). Thus, the proliferation of CD8^+^ T cells was decreased by coculture with IL-10R2^+^IL-22R1^−^ or IL-10R2^+^IL-22R1^+^ myeloid cells compared to those cocultured with IL-10R2^−^IL-22R1^−^ myeloid cells (Fig. 3h, left). Additionally, granzyme B expression in CD8^+^ T cells was decreased by coculture with IL-10R2^+^IL-22R1^−^ and IL-10R2^+^IL-22R1^+^ myeloid cells (Fig. 3h, right). Collectively, IL-10R2^+^/IL-22R1^+^ myeloid cells inhibited immune activation, contributing to the nonimmunogenic nature of TME in promoting PDAC progression.

### Clinical significance of IL-10R2^+^ myeloid cells for the early detection of PDAC and its recurrence

**Fig. 4:**
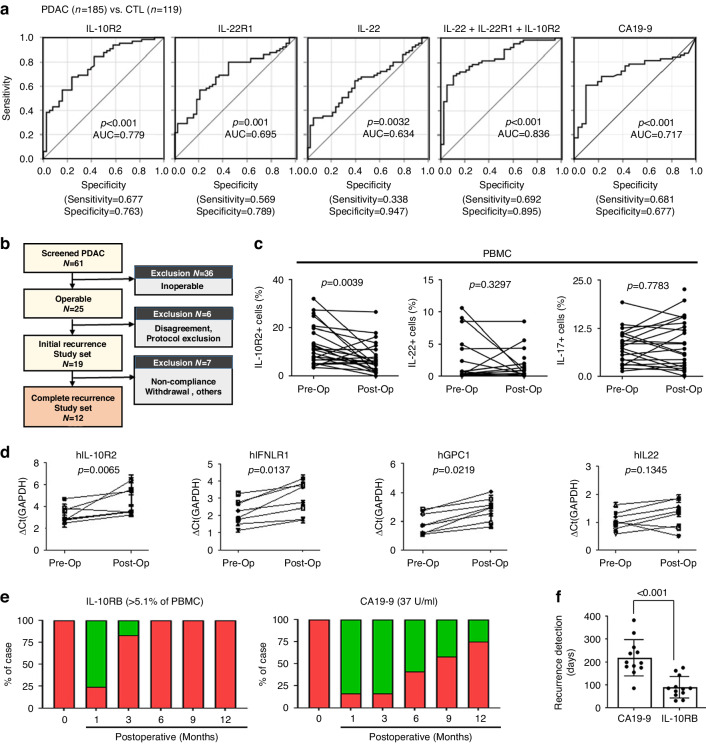
Clinical significance of IL-10R2^+^ population in PDAC PBMCs for early detection and recurrence after pancreatectomy. **a** Receiver operating characteristic (ROC) curve for IL-10R2, IL-22R1, IL-22, combination of IL-22^+^IL-22R1^+^IL-10R2 and CA19-9 in PBMCs from PDAC patients (*n* = 180) vs. controls (CTL, *n* = 98). Multivariable logistic regression was used to determine the independent predictors of PDAC: variables showing *p*-values < 0.05 by univariable logistic regression were entered. A combination of IL-10R2, IL-22R1 and IL-22 for PDAC was compared using the DeLong method. **b** Schematic illustration of the study flow and sample size. **c** Levels of IL-10R2^+^, IL-22^+^ and IL-17^+^ cells in PBMC from the pre- and postoperative (1 month) samples (paired *t*-test). **d** ∆Ct values of IL-10R2, IFNLR1, GPC1 and IL-22 were measured by qPCR and compared between pre-and postoperative (1 month) samples (paired *t*-test). **e** A fraction of biomarkers (IL-10R2 or CA19-9) detected among 12 radiologically confirmed recurrence cases in a time-dependent manner (red: over the cut-off value; green: under the cut-off value). **f** Comparison of the median number of days until tumour recurrence detection after surgery for each biomarker (Mann-Whitney U test).

Increased IL-10R2^+^ or IL-22R1^+^ cells in the peripheral blood of PDAC patients might represent an effective diagnostic marker. Patients with high IL-10R2 or IL-22R1 expression had a lower survival rate than patients with low IL-10R2 or IL-22R1 expression (*p* = 0.003 or 0.00008, respectively; Figure S5, in Additional file 4), indicating an inverse relationship between IL-10R2 or IL-22R1 expression and patient survival. To evaluate IL-10R2^+^ or IL-22R1^+^ cells as potential diagnostic biomarkers of PDAC, we determined the clinical significance and value of IL-10R2 and IL-22R1 expression in peripheral blood cells for PDAC detection by measuring the sensitivity and specificity of positive mRNA expression in patients through a hospital-based prospective study. Other IL-10 family cytokine receptors (e.g. IFNLR, IL-10R1, IL-20R1 and IL-22R2) were also detected during the analysis. The sensitivity and specificity were 0.677 and 0.763 for IL-10R2 mRNA, respectively (AUC = 0.779, *p* < 0.0001), 0.569 and 0.789 for IL-22R1, respectively (AUC = 0.695, *p* < 0.001), 0.338 and 0.947 for IL-22, respectively (AUC = 0.634, *p* = 0.0032) and 0.681 and 0.677 for CA-19-9, respectively (AUC = 0.717, *p* < 0.001) (Fig. 4a). As determined by logistic regression, the combination of IL-22, IL-22R1 and IL-10R2 showed significant improvement in the AUC (0.836) with a sensitivity of 0.692 and specificity of 0.895 (*p* < 0.0001) (Fig. 4a). The sensitivity and specificity values were similar between mRNA (as determined by qPCR) and protein analyses (cell frequencies were determined by FACS analysis, data not shown). However, the sensitivity and specificity were not improved by adding IFNLR, IL-10R1 or other receptor mRNA levels to the aforementioned combination (data not shown). Therefore, the IL-10R2^+^ myeloid population size in PBMCs alone was positively correlated with PDAC detection, and this correlation was more potent when combined with IL-22R1^+^ and IL-22^+^ populations.

In addition to tumour detection, the availability of early markers of recurrence after surgery is invaluable. Accordingly, we compared the expression of IL-10R2^+^ myeloid cells from PDAC patients who underwent a margin-free pancreas resection. Initially, 61 patients who received surgery were recruited, and recurrence was confirmed by MRI or CT in 12 cases who completed monthly analysis (Fig. 4b). Preoperatively upregulated IL-10R2^+^ cells were significantly reduced or absent at 1 month after surgery (Fig. 4c), comparable to the results for CA19-9 that was previously reported as a recurrence indicator for PDAC [42] (data not shown). In contrast, IL-22^+^ and IL-17^+^ cells did not show different expression levels between pre- and postoperative states (Fig. 4c). Moreover, the mRNA level of IL-10R2 in all PBMCs was significantly reduced after surgery (Fig. 4d). As with IL-10R2, the initial reduction in IFNLR1 and hGPC1 was observed 1 month after pancreatectomy (Fig. 4d). However, neither IFNLR1 nor hGPC1 correlated with tumour recurrence until 12 months after surgery, unlike IL-10R2. Additionally, from serial observations and comparisons of IL-10R2 and CA19-9 in recurrent PDAC, an earlier elevation of IL-10R2 was detectable compared to CA19-9 (Fig. 4e, f). Six months after surgery, all recurrence cases exhibited significantly elevated IL-10R2 even when the recurrent mass was not yet evident on an abdominal CT. Additionally, 7 cases (58.3%) even showed normal CA19-9 levels. At 9 months, whilst all recurrence cases continuously showed elevated IL-10R2^+^ blood cells, 4 patients (33.3%) with recurrence still exhibited normal CA19-9 levels. Moreover, the mean time to CA19-9 elevation after surgery was 239.7 ± 125.2 days, whereas it was 109.9 ± 41.1 days for IL-10R2 (*p* < 0.0001, paired t-test) (Fig. 4f), suggesting that increased IL-10R2^+^ myeloid cells might be a useful indicator of PDAC recurrence. Therefore, increased IL-10R2^+^ myeloid cells in PDAC patients can be used to diagnose PDAC early and detect tumour recurrence after pancreatectomy.

## Discussion

We initially hypothesised that each cancer entity harbours specific circulating immune cells that migrate into the tumour, participate in establishing the TME and actively interact with tumour cells. This hypothesis is based on the “cancer immunoediting” concept, where innate or adaptive immune cells can monitor, detect and react with tumours and might participate in tumour elimination, especially in the early period [43]. Supporting this idea, a recent study reported that macrophages alter their transcriptome for specific cancer subtypes [31]. In PDAC, CSF1R^+^F4/80^+^ macrophages exclusively infiltrate the tumour and enhance its growth [44]. Although myeloid cells in cancer tissue originate from the bone marrow and migrate through the peripheral circulation to reach the target tumour, most previous studies focused only on the characteristics and function of tumour-infiltrating myeloid cells and not on changes in the blood cell subtypes or their specific markers.

IL-22 is typically produced by immune cells, mainly innate immune cells and some lymphoid lineages [13]. Its receptors are usually expressed in normal tissues, such as IL-22R1 in the intestinal and skin epithelium [13, 14]. However, the relationship between the IL-22 ligand and the expression patterns of its receptors is reversed in PDAC carcinogenesis. Although IL-22 is not detected in the normal pancreas, we found that PDAC could actively produce this cytokine, similar to previous studies [11, 12, 20]. IL-22 is a cancer-promoting cytokine that is elevated in various cancers [45, 46]. In this research, IL-22 expression was elevated in PDAC (Fig. 1a, S1). Recent studies revealed that IL-22 enhances immune cell infiltration in the tumour and increases malignancy [46, 47]. In this context, enhanced IL-22 expression could recruit immune cells expressing IL-22R, and the recruited immune cells promote further PDAC progression. Since we found myeloid cells expressing the IL–22–binding receptor (e.g. IL-10R2^+^CD45^+^) in PDAC, we attempted to identify these cells in the peripheral blood of PDAC patients. As IL-22 has two heterodimeric receptors, IL-10R2 and IL-22R1, we initially attempted to assess IL-10R2^+^IL-22R1^+^ DPCs in the peripheral blood of PDAC patients. However, a few DPCs were found in the blood of most of our PDAC patients. Additionally, unlike IL-10R2^+^CD45^+^ cells, which were found in most PDAC patients (92.4%, 244/264), IL-22R1^+^CD45^+^ cells were found in a comparatively lower number of patients (15.6%, 45/264). However, IL-10R2^+^IL-22R1^+^ cells were abundant in PDAC in humans (Fig. 2h) and mouse models (Fig. 3e, f). Despite no direct evidence for converting IL-10R2^+^ myeloid cells into DPCs, DPCs might be generated by PDAC-specific TME. Moreover, these cells might not egress into the peripheral circulation. Therefore, elevated IL-22 expression in PDAC might induce the recruitment and accumulation of immune cells from the circulation. However, further studies on the detailed molecular mechanism of this increase in IL-10R2^+^ cells are needed.

The AUC value showed a stronger correlation with IL-10R2 than IL-22R1, and the correlation with the combination of the three markers was much stronger than that for each single marker alone (Fig. 4a). Thus, IL-10R2^+^ and IL-22R1^+^ myeloid cells might be independently generated and separately contribute to the PDAC immune environment, thereby working synergistically to improve PDAC detection by enhancing the AUC value. In our murine PDAC model data, IL-10R2^+^ cells reflected tumour progression (Fig. 3). However, IL-22R1^+^ cells temporarily increased in the circulation and tumour. These data support the broad and effective use of IL-10R2 as a predictive marker. However, further studies on the differential regulation of IL-10R2 and IL-22R1 expression in PDAC progression are required.

Previous studies reported that some malignancies and inflammatory conditions can increase IL-22R1^+^ lymphocytes and granulocytes, respectively [48, 49]. Additionally, the aberrant expression of IL-22R1 in blood cells aggravated inflammatory conditions in a mouse model [50]. However, why IL-22R1^+^ myeloid cells emerge to a lesser extent than IL-10R2^+^ myeloid cells in the peripheral blood of PDAC patients remains uncertain. Since most PDAC patients had IL-10R2^+^ cells in their blood, we investigated their relationship with cancer progression. Besides PDAC tumour, CoCa and LC express IL-22 [15, 16]. Although we could not detect IL-10R2^+^ blood cells in PBMCs of these tumour types (data not shown), IL-10R2^+^ cells might have increased in early tumorigenesis in each cancer, with IL-22 level being high in tumour tissue, and tumour-associated myeloid cells playing an important role. In this study, IL-10R2^+^ cells were not detectable in either benign pancreatic or peripancreatic diseases, such as CP or liver cirrhosis (Fig. 1c).

The high mortality of PDAC is mainly caused by the lack of early detection and curative late-stage therapy [1, 2]. An early diagnostic method could be useful in clinical trials for new therapeutic reagents or strategies, especially in PDAC [51]. Although CA19-9 and DUPAN-2 can be used to monitor PDAC patients, these markers are not useful for screening and diagnosing, especially for early cases [52, 53]. Hence, extensive research has focused on identifying new biomarkers based on biological, immunological and genetic changes using blood, urine [54] and saliva [55] samples. Compared to previous reports, we focused on cancer-specific immune cells but not serum factors that are often unidentifiable regarding their origins. Previously, most marker screening studies used serum or tumour tissues. However, in these methods, the detection threshold depends on the mass size: the marker level increases as the tumour mass grows. Thus, the detection methods are limited for an early lesion with a small mass. However, the levels of immune cell markers do not depend on the tumour mass. Therefore, using these cells as disease-specific biomarkers could be valuable, especially for detecting small lesions. Hence, several specific immune cells were successfully identified and found to be specific to PDAC compared to other tumours. Besides IL-10R2 or IL-22R1, IL-10R2^+^ myeloid cells can express other specific markers following PDAC progression. Some markers noted by us were previously identified. CSF1R was highly expressed in IL-10R2^+^ cells. Furthermore, CSF1R^+^ macrophages contribute to pancreatic cancer growth through T cell suppression [44]. Additionally, *CX3CR1* was identified in our study. Marchesi et al. [56] and found that *CX3CR1* is expressed in PDAC, observing that the CX3CR1-CX3CL1 axis contributes to tumour relapse. Romero et al. [57] reported that several chemokines are associated with T-cell infiltration of PDAC tissue. Consistently, we detected enhanced expression of *CXCL9* and *CXCL10* in IL-10R2^+^ cells, which might participate in the immunosuppression of PDAC. Thus, future human and in vivo studies on these cells and other PDAC-specific immune cell markers, combined with IL-10R2, will expand the list of effective markers for PDAC and their detection range.

Regarding PDAC recurrence after cancer surgery (Fig. 4e), IL-10R2^+^ cells significantly decreased or disappeared immediately after surgery and reappeared upon tumour recurrence early during tumour growth. The emergence of IL-10R2^+^ cells was detectable 130 days sooner than that of CA19-9 in predicting tumour recurrence (*p* < 0.001, Fig. 4f), with imaging studies confirming this result (CT or MRI, data not shown). Moreover, in an incidentally identified case with an extremely small tumour (6.3 mm), the patient showed increased IL-10R2^+^ cells despite normal CA19-9 and negative imaging data. Therefore, IL-10R2^+^ myeloid cells might effectively indicate PDAC development or recurrence before CA19-9 elevation or a positive imaging result, possibly due to its role in early cancer nonimmunogenic TME development. Although we identified elevated IL-10R2^+^ cells and typical mRNA signatures in our PDAC patients, cases showing recurrence and a mouse model of pancreatic cancer, a large-scale population-based prospective study is needed to confirm the clinical utility of these cells for screening the general population. Additionally, although we found that IL-10R2^+^/IL-22R1^+^ myeloid cells increased in TME and inhibited immune activation in TME in vitro and in situ, the mechanisms underlying the development of IL-10R2^+^IL-22R1^+^ cells and their functional roles were not fully determined. Hence, future studies should also investigate the mechanisms underlying the generation of these cells and their migration to tumour tissues.

In conclusion, IL-10R2^+^IL-22R1^+^ cells are novel markers for PDAC. These markers have potential applications for general screening and early diagnosis, which will significantly aid the development of new treatment strategies for PDAC. Additionally, advanced omics and exosome analysis techniques can be used to precisely investigate heterogeneous IL-10R2^+^ myeloid cells regarding their activation period, functions and interactions with tumour cells as IL-10R2^+^ myeloid cells infiltrating the tumour tissues are highly correlated with tumour stage and survival in PDAC.

### Supplementary information

Table S1

Table S2

Table S3

Supplementary figures

## Data Availability

scRNA-Seq data generated in this study are available on Gene Expression Omnibus (GEO) with accession number PRJNA917457. Almost all data generated or analysed during this study are included in this manuscript and additional files, while others are available from the corresponding author on reasonable request, except for the information related to patient privacy.

## Code availability

The source codes used in the scRNA-seq analysis are available on GitHub at: https://github.com/CB-postech/scRNAseq_of_PDAC_PBMC.
