# Supplementary material for: Tumour-associated myeloid cells expressing IL-10R2/IL-22R1 as a potential biomarker for diagnosis and recurrence of pancreatic ductal adenocarcinoma
Source: Br J Cancer. 2024 Apr 20;130(12):1979–89. doi: 10.1038/s41416-024-02676-w (PMC11183123; doi:10.1038/s41416-024-02676-w)
Supplement: Supplementary file 1 — Table S1 [file 41416_2024_2676_MOESM1_ESM.docx]

**Table S1**. **PDAC Patient Characteristics.**

The table provides a comprehensive overview of patient characteristics in the study cohort. Demographic details include a near-even distribution between male (51.9%) and female (48.1%) participants, with a median age of 69 years. Lifestyle factors indicate that 25.3% were smokers, 31.8% consumed alcohol, and 37.0% had diabetes. The median BMI is 23. Medical features include an initial mean CA19-9 level of 2149.2 U/ml. Pancreatic mass locations vary, with the majority in the head (35.1%). Resectability status indicates that 35.7% were resectable, while 64.3% were unresectable. Initial treatments comprised surgery (27.9%), chemotherapy (59.7%), and no treatment (12.3%). Disease stages varied, with 5.2% in Stage I, 21.4% in Stage II, 33.1% in Stage III, and 40.3% in Stage IV. The table provides a clear and structured snapshot of patient characteristics, crucial for understanding the diverse aspects of the study cohort.

| **Sex** | **Male, N (%)** | 80 (51.9) |
| --- | --- | --- |
|  | **Female, N (%)** | 74 (48.1) |
| **Age** | **Years, median** | 69 |
| **Smoking** | **Yes, N (%)** | 39 (25.3) |
|  | **No, N (%)** | 115 (74.7) |
| **Alcohol** | **Yes, N (%)** | 49 (31.8) |
|  | **No, N (%)** | 105 (68.2) |
| **DM** | **Yes, N (%)** | 57 (37.0) |
|  | **No, N (%)** | 97 (63.0) |
| **BMI** | **median** | 23 |
| **Initial CA19-9** | **U/ml, mean (SD)** | 2149.2 (±4491.1) |
| **Location of main mass** | **Head, N (%)** | 54 (35.1) |
|  | **body, N (%)** | 31 (20.1) |
|  | **Tail, N (%)** | 15 (9.7) |
|  | **Uncinate, N (%)** | 20 (13.0) |
|  | **Neck, N (%)** | 6 (3.9) |
|  | **Overlap, N (%)** | 28 (18.2) |
| **Resectability** | **Resectable** | 55 (35.7) |
|  | **Unresectable** | 99 (64.3) |
| **Initial treatment** | **Surgery** | 43 (27.9) |
|  | **Chemotherapy** | 92 (59.7) |
|  | **No treatment** | 19 (12.3) |
| **Stages** | **I** | 8 (5.2) |
|  | **II** | 33 (21.4) |
|  | **II** | 51 (33.1) |
|  | **iV** | 62 (40.3) |
