# Supplementary material for: Tumour-associated myeloid cells expressing IL-10R2/IL-22R1 as a potential biomarker for diagnosis and recurrence of pancreatic ductal adenocarcinoma
Source: Br J Cancer. 2024 Apr 20;130(12):1979–89. doi: 10.1038/s41416-024-02676-w (PMC11183123; doi:10.1038/s41416-024-02676-w)
Supplement: Supplementary file 2 — Table S2 [file 41416_2024_2676_MOESM2_ESM.docx]

**Table S2**. **Antibodies and reagents used in this study.**

| **Reagents and Antibodies** | **Cat. No** | **Company** |
| --- | --- | --- |
| Anti-IL-22R1 antibody | ab211675 | Abcam |
| Anti-IL-10R2 antibody | ab106282 | Abcam |
| Purified anti-CD45 antibody | 610266 | BD |
| Fluorescein (FITC) AffiniPure F(ab')₂ Fragment Goat Anti-rabbit IgG (H+L) | 111-096-144 | Jackson |
| Alexa Fluor® 594 AffiniPure F(ab')₂ Fragment Donkey Anti-mouse IgG (H+L) | 715-586-150 | Jackson |
| APC AffiniPure F(ab')₂ Fragment Goat Anti-Rat IgG (H+L) | 112-136-072 | Jackson |
| Anti-human IL-10 antibody | ab34843 | Abcam |
| Anti-human IL-17 antibody | ab79056 | Abcam |
| Anti-human IL-20 antibody | ab229809 | Abcam |
| Anti-human IL-22 antibody | ab18498 | Abcam |
| Anti-human IL-23 antibody | ab45420 | Abcam |
| Anti-human IL-26 antibody | ab254476 | Abcam |
| Anti-mouse CD8 antibody | ab217344 | Abcam |
| EnVision+/HRP, Rabbit | K400311-2 | DAKO |
| PE anti-human IL-22 antibody | 66704 | Biolegend |
| Percp anti-human IL-22R1 antibody | FAB2770C | R&D |
| PE anti-human IL-10R2 antibody | 308804 | Biolegend |
| FITC anti-human IL-17 antibody | 512304 | Biolegend |
| PE anti-human IL-10R1 antibody | 556013 | BD |
| Percp anti-mouse IL-22R1 antibody  PE anti-mouse IL-22R1 antibody  APC anti-mouse IL-10R2 antibody | FAB42941C  FAB42941P  FAB53681A | R&D  R&D  R&D |
| PE anti-mouse IL-10R2 antibody | 112706 | Biolegend |
| APC anti-mouse/human CD11b antibody  Percp/Cy5.5 anti-mouse CD45 antibody | 101212  45-0451-82 | Biolegend  Invitrogen |
| FITC anti-mouse CD49b antibody | 108906 | Biolegend |
| FITC anti-mouse CD3 antibody | 100306 | Biolegend |
| PE/Cy7 anti-mouse CD8 antibody | 100722 | Biolegend |
| TruStain FcX anti-mouse CD16/32 antibody  Zombie aqua fixable viability kit  Anti-mouse/human IL-10R2 antibody | 101320  423101  MAB874 | Biolegend  Biolegend  R&D |
| Anti-mouse/human IL-10R2 antibody | MM0360-8R24 | Novusbio. |
| Histopaque-1077 | 10771 | Sigma |
| Histopaque-1119 | 11191 | Sigma |
| Histopaque-1083 | 10831 | Sigma |
| Collagenase type XI | C7657 | Sigma |
| Dispase I | D4818 | Sigma |
| Dispase II  DNase I  RBC Lysis Buffer | D4693  10104159001  00-4333-57 | Sigma  Roche  ThermoFisher |
| DRAQ7(dead cell stain dye, far-red emitting) | 424001 | Biolegend |
| Qiazol lysis reagent | 79306 | Qiagen |
| Nuclease-Free Water | AM9938 | Invitrogen |
| Prime script RT master mix | RR036A | Takara |
| SYBR Premix Ex taq | RR420A | Takara |
| DMEM | 10-566-016 | Gibco |
| RPMI 1640 | 72400120 | Gibco |
| FBS | 16000044 | Gibco |
| Penicillin-streptomycin, liquid | 15070063 | Gibco |
| TrypLE™ Express | 12604013 | Gibco |
| 1x PBS | 10010-049 | Gibco |
| BSA | A3311 | Sigma |
| Trypan blue stain, 0.4% | 17-942E | Lonza |
| Dako REAL Peroxidase-Blocking Solution | S202386-2 | DAKO |
| Target Retrieval Solution, Concentrated x 10 | S236784-2 | DAKO |
| Antibody diluent with background-reducing | S302283 | DAKO |
| Real envision detection systems | K500711 | DAKO |
| Hematoxylin, Mayer's | S330930 | DAKO |
| Vectashield mounting medium w DAPI | H-1200 | Vector |
| Giemsa stain kit | ab150670 | Abcam |
| Pico sirius red staining kit | ab150681 | Abcam |
| Trichrome stain kit  EasySep Mouse CD11b Positive Selection Kit II  EasySep Mouse T cell Isolation Kit  Ultra-LEAF Purified anti-mouse CD3 Antibody  Ultra-LEAF Purified anti-mouse CD28 Antibody  CellTrace CFSE Cell Proliferation Kit  Recombinant Human IL-2  Human PBMNC  Recombinant Human GM-CSF  Recombinant Human IL-6  Corning Costar 96-Well, Cell culture treated U-shaped-Bottom Microplate | ab150686  18970  19851  100238  122022  C34554  200-02  70025  300-03  200-06  07-200-95 | Abcam  STEMCELL  STEMCELL  Biolegend  Biolegend  ThermoFisher  Peprotech  STEM CELLS  Peprotech  Peprotech  Corning |
