# Supplementary material for: Tumour-associated myeloid cells expressing IL-10R2/IL-22R1 as a potential biomarker for diagnosis and recurrence of pancreatic ductal adenocarcinoma
Source: Br J Cancer. 2024 Apr 20;130(12):1979–89. doi: 10.1038/s41416-024-02676-w (PMC11183123; doi:10.1038/s41416-024-02676-w)
Supplement: Supplementary file 3 — Table S3 [file 41416_2024_2676_MOESM3_ESM.docx]

**Table S3. Primers used in the qRT-PCR assays**

| **Gene** | **Primer** | **Sequence** |
| --- | --- | --- |
| *hIL22* | forward | 5'-AAC TTC CAG CAG CCC TAT ATC-3' |
|  | reverse | 5'-ATA GCA GCG CTC ACT CAT AC-3' |
| *hIL22R1* | forward | 5'-CTG GGA CAC TTT CTA GTC CTA AAC C-3' |
|  | reverse | 5'-GCT TCT TGG GAT TCC TCC ATA G-3' |
| *hIL10R2* | forward | 5'-ATG AGC ATT CAG ACT GGG TAA A-3' |
|  | reverse | 5'-GGG CTA AGA AAC GCA TAT GTA AAG-3' |
| *hIFNLR1* | forward | 5'-CGT TCA GTG TCC CGA AAT ACA -3' |
|  | reverse | 5'-CCC TGC GGC AAT TAC TAA CA-3' |
| *hGPC1* | forward | 5'-GTC ATG AAG CTG GTC TAC TG-3' |
|  | reverse | 5'-AGC CCT TGA GCA CAT TTC-3' |
| *hIL29* | forward | 5'-CTA GAC CAG CCC CTT CAC AC-3' |
|  | reverse | 5'-AAG GTG ACA GAT GCC TCC AG-3' |
| *hIL20R1* | forward | 5'-CTG AAA GTG GAC GGT TCT ATC-3' |
|  | reverse | 5'-GCT GTC AGG ACA ACA GAA A-3' |
| *hIL20R2* | forward | 5'-CCT GAA GCA TCC CTT TAA TAG A-3' |
|  | reverse | 5'-AGG TCC TCC AGC TCA ATA A-3' |
| *hIL10R1* | forward | 5'-GTC ATC ATC TTC TTT GCC TTT G-3' |
|  | reverse | 5'-GAC ACT GGG TAG CTT CTT TC-3' |
| *hIL17Ra* | forward | 5'-GCG TCA GGT TTG AGT TTC T-3' |
|  | reverse | 5'-GTG AAC GGT CAC CTC ATA TTC3' |
| *hIL17Rc* | forward | 5'-CTC TGT GGT ATA TGA CTG CTT C-3' |
|  | reverse | 5'-GTG TGG TTG AGT TCC TTC TC-3' |
| *hIL-7R* | forward | 5'- GTA GTC ATC ACT CCA GAA AGC-3' |
|  | reverse | 5'-ACC TGG AAG AGG AGA GAA TAG-3' |
| *hFLT3LG* | forward | 5'-TGG AGC CCA ACA ACC TAT CT-3' |
|  | reverse | 5'-TAG TCA GAC AGC TCA CGG ATT T-3' |
| *hCD27* | forward | 5'-GAA GGA CTG TGA CCA GCA TAG A-3' |
|  | reverse | 5'-CGA ACG AGA AGA CCA GAG TTA CA-3' |
| *hGAPDH* | forward | 5'-CAT GTT CGT CAT GGG TGT GAA CCA-3' |
|  | reverse | 5'-AGT GAT GGC ATG GAC TGT GGT CAT-3' |
