# Supplementary material for: Tumour-associated myeloid cells expressing IL-10R2/IL-22R1 as a potential biomarker for diagnosis and recurrence of pancreatic ductal adenocarcinoma
Source: Br J Cancer. 2024 Apr 20;130(12):1979–89. doi: 10.1038/s41416-024-02676-w (PMC11183123; doi:10.1038/s41416-024-02676-w)
Supplement: Supplementary file 4 — Supplementary figures [file 41416_2024_2676_MOESM4_ESM.docx]

**
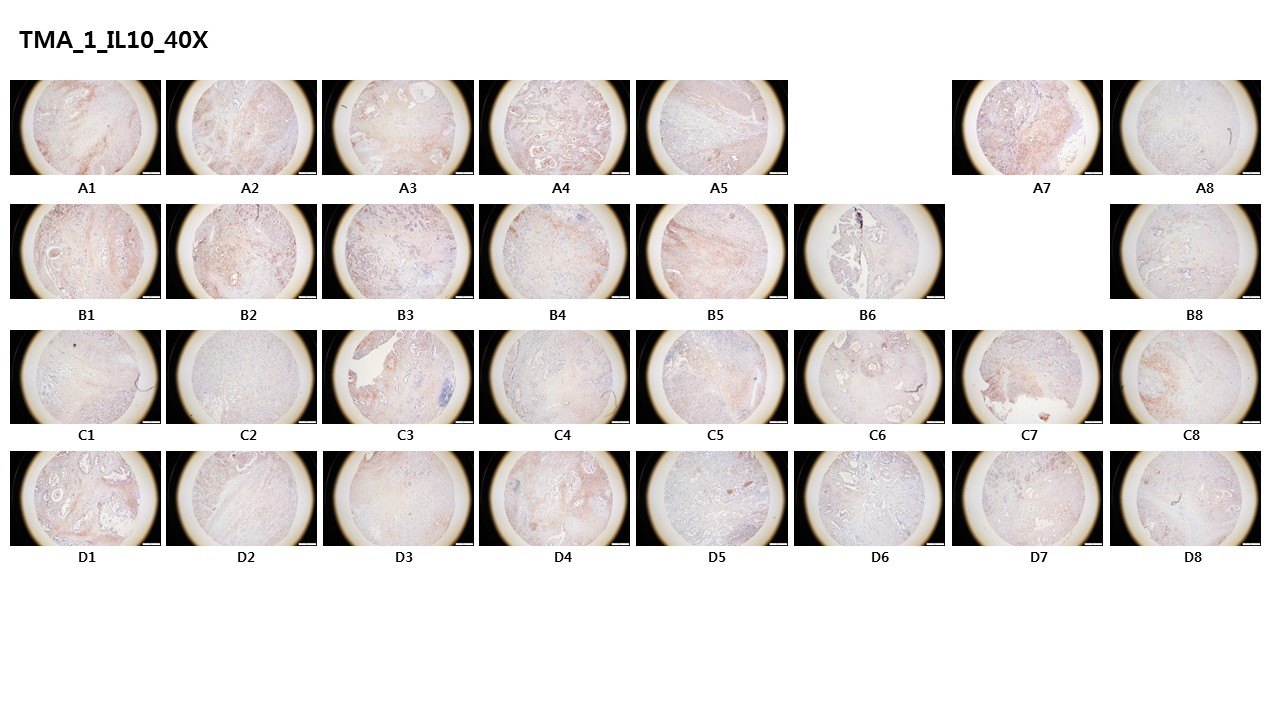
**

**
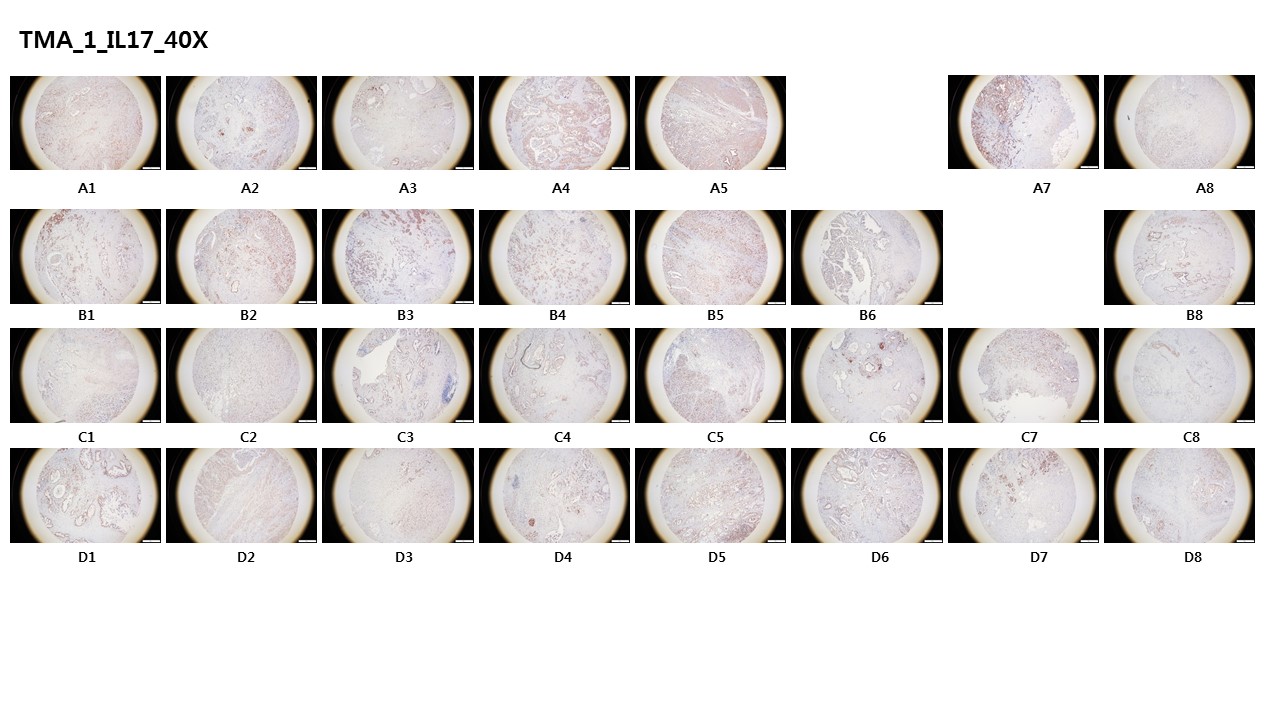

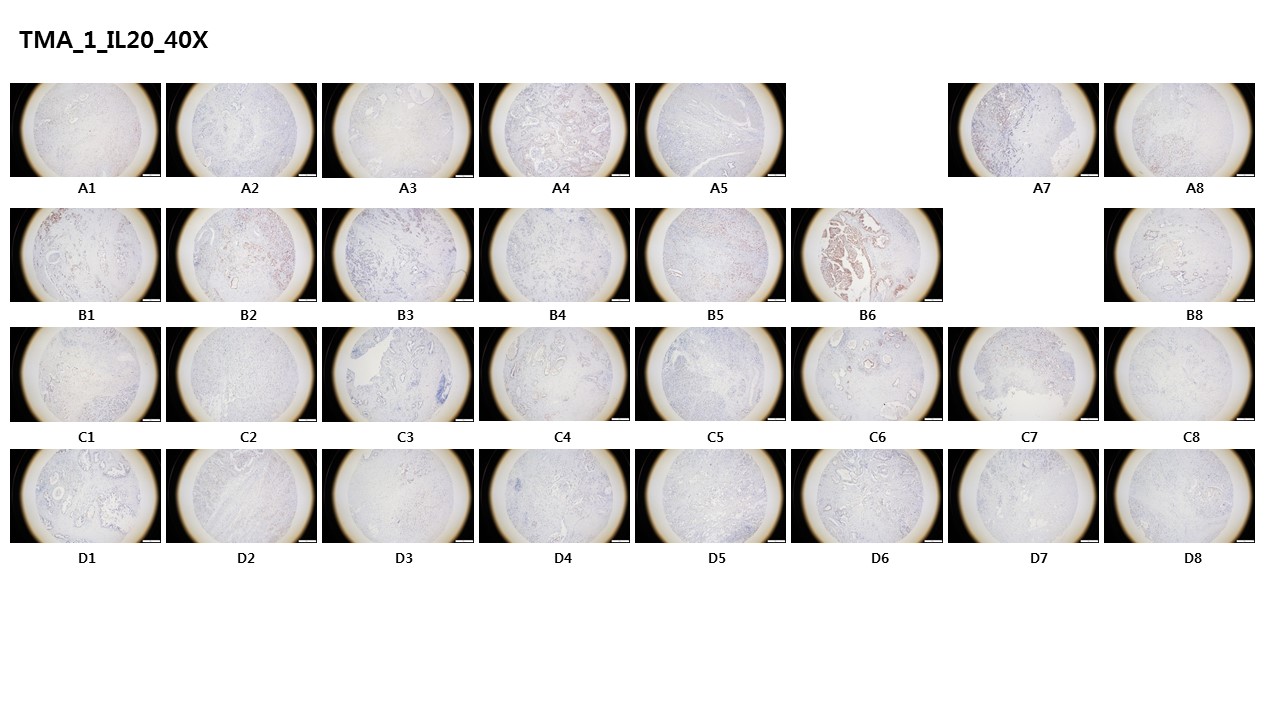

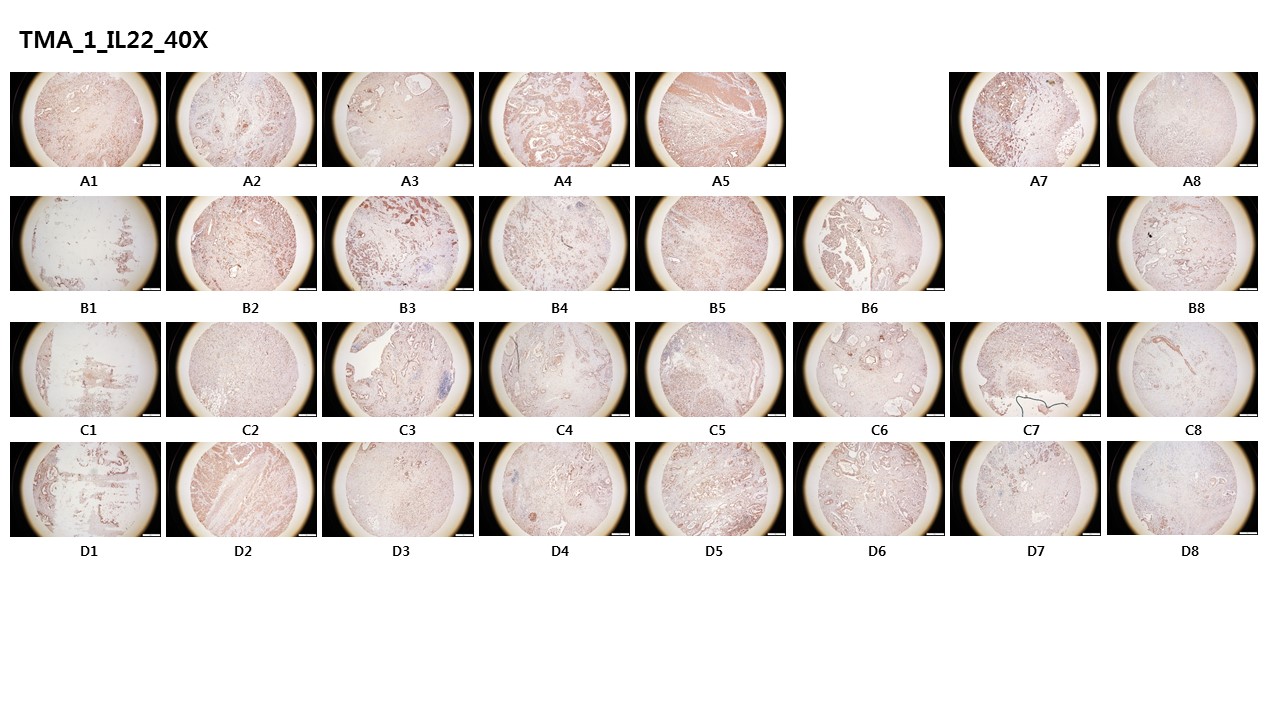

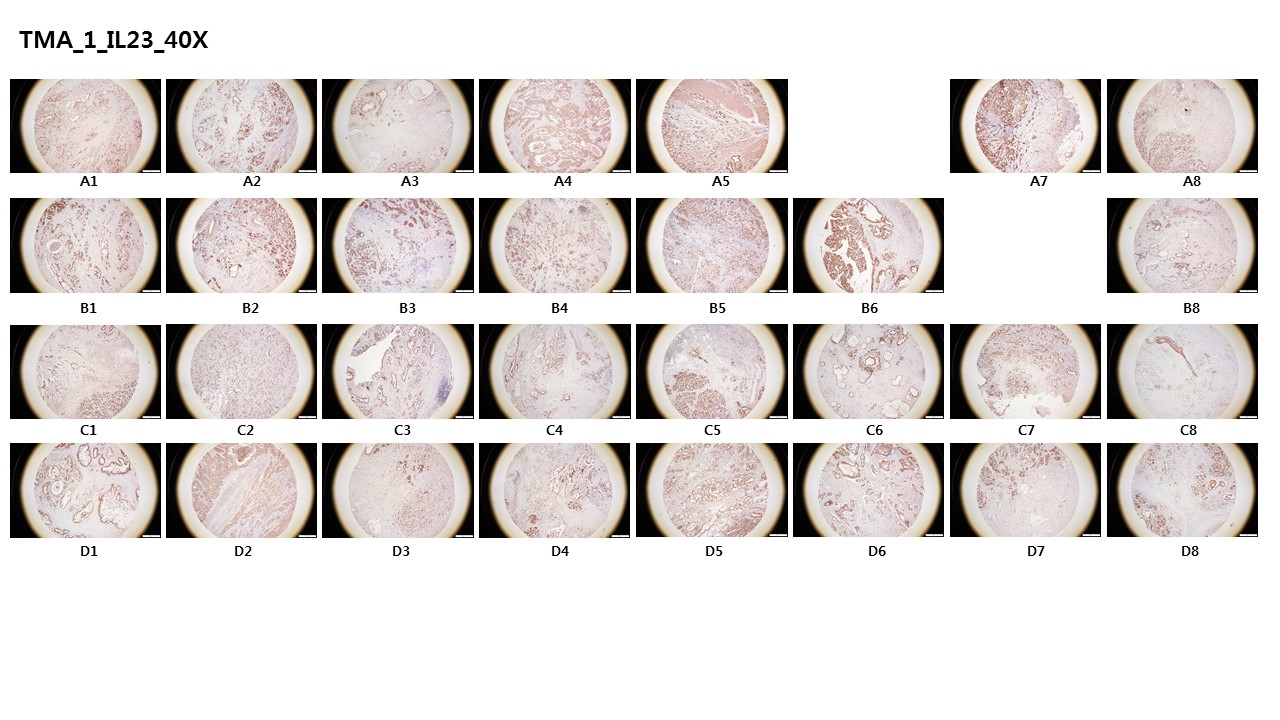
**

**
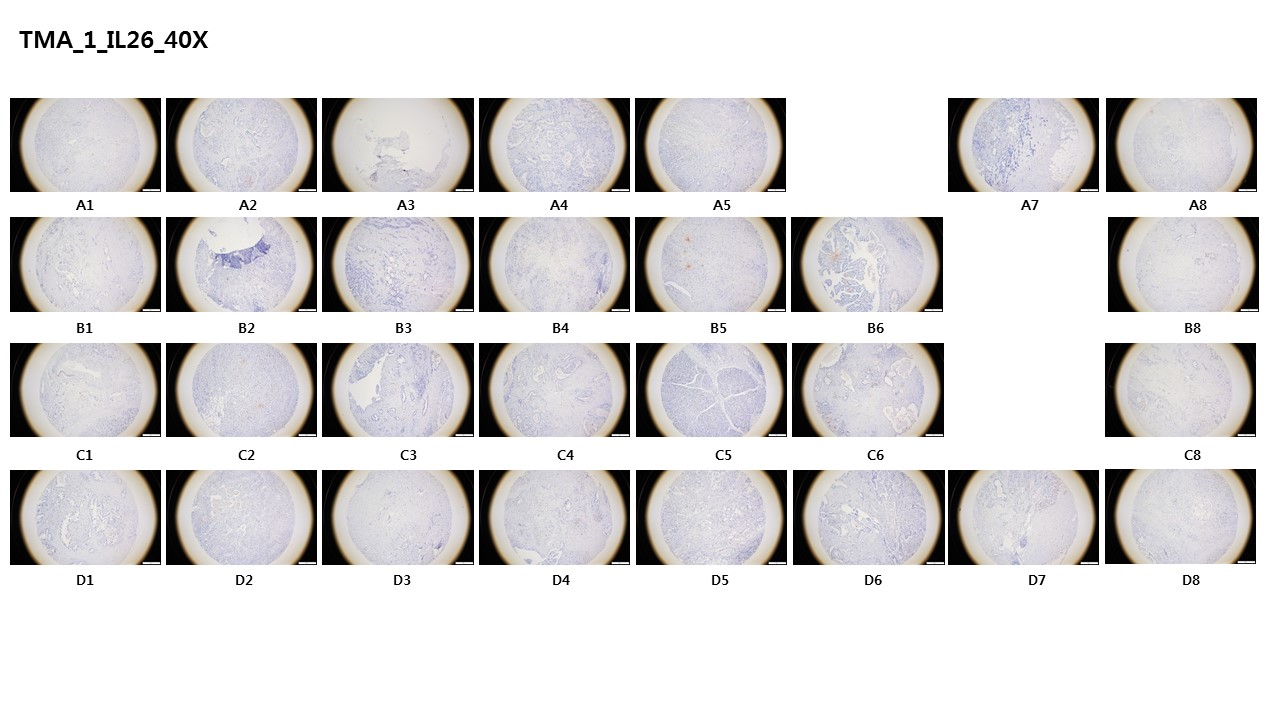
**

**Figure S1. Representative immunohistochemical staining of IL-10 family cytokines on a TMA from a PDAC patient** **(n=29).** The full images from microscope were presented.


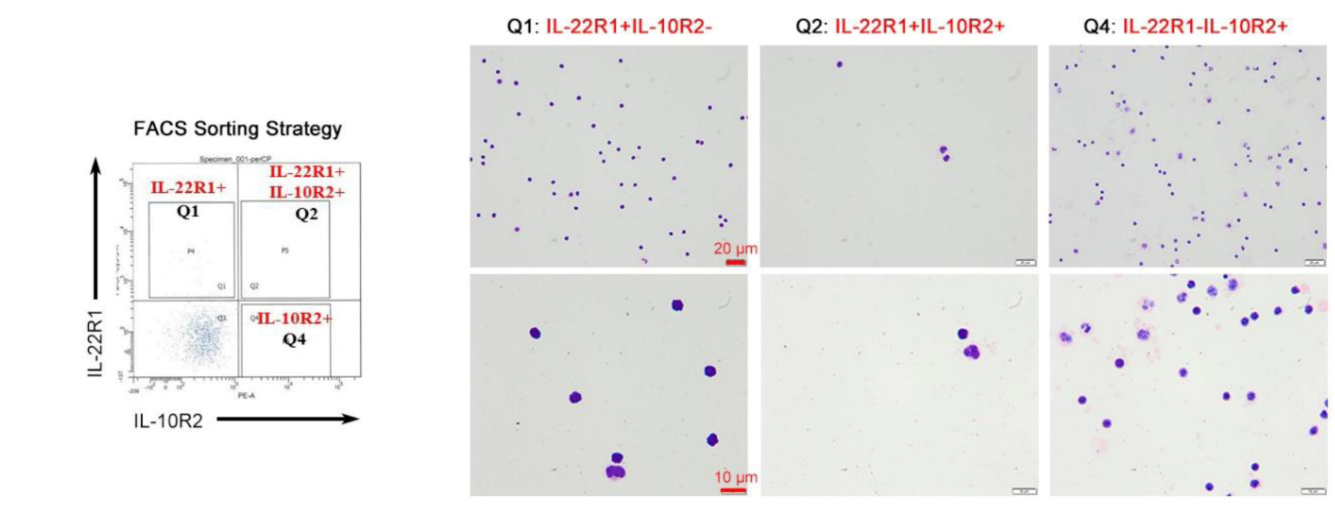


**Figure S2. Characterization of IL-10R2+ or IL-10R2- cells from PDAC patients.** Representative images of IL-10R2+ or IL-22R1+ cells after Giemsa staining.


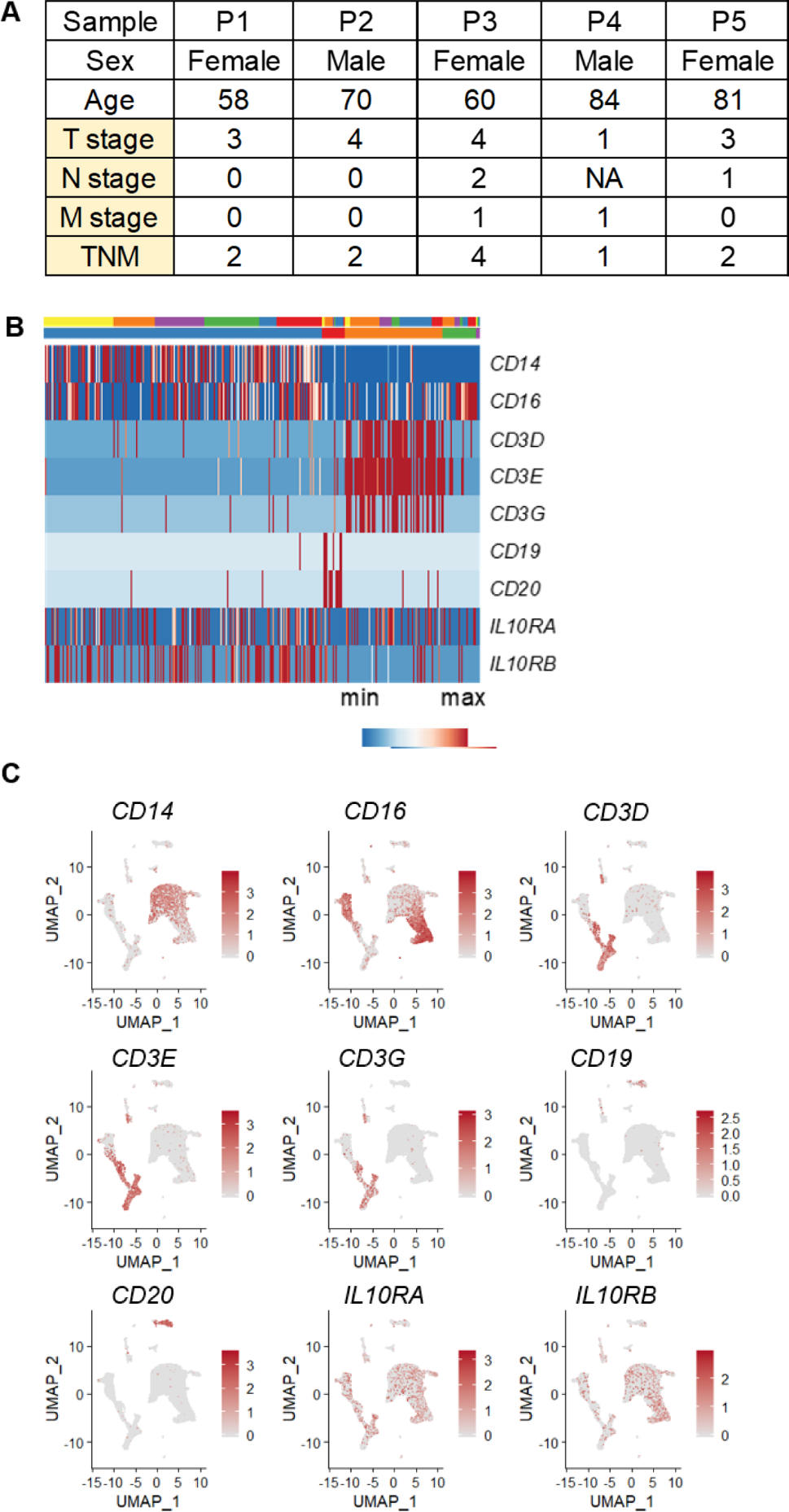


**Figure S3. Characterization of the immune types of IL-10R2+ or IL-10R2- cells from PDAC patients.** (**A**) Summary of patient metadata. (**B**) Heatmap showing the relative expression (Z-score) of the indicated cell type marker genes for monocytes/macrophages (CD14), monocytes/NK cells (CD16), B cells (CD19 and CD20), and T cells (CD3D, CD3E, and CD3G). The IL-10 receptor genes (IL10R1 and IL10R2) are included to show the upregulation of both genes. (**C**) UMAP plots showing the expression levels of the indicated marker genes.


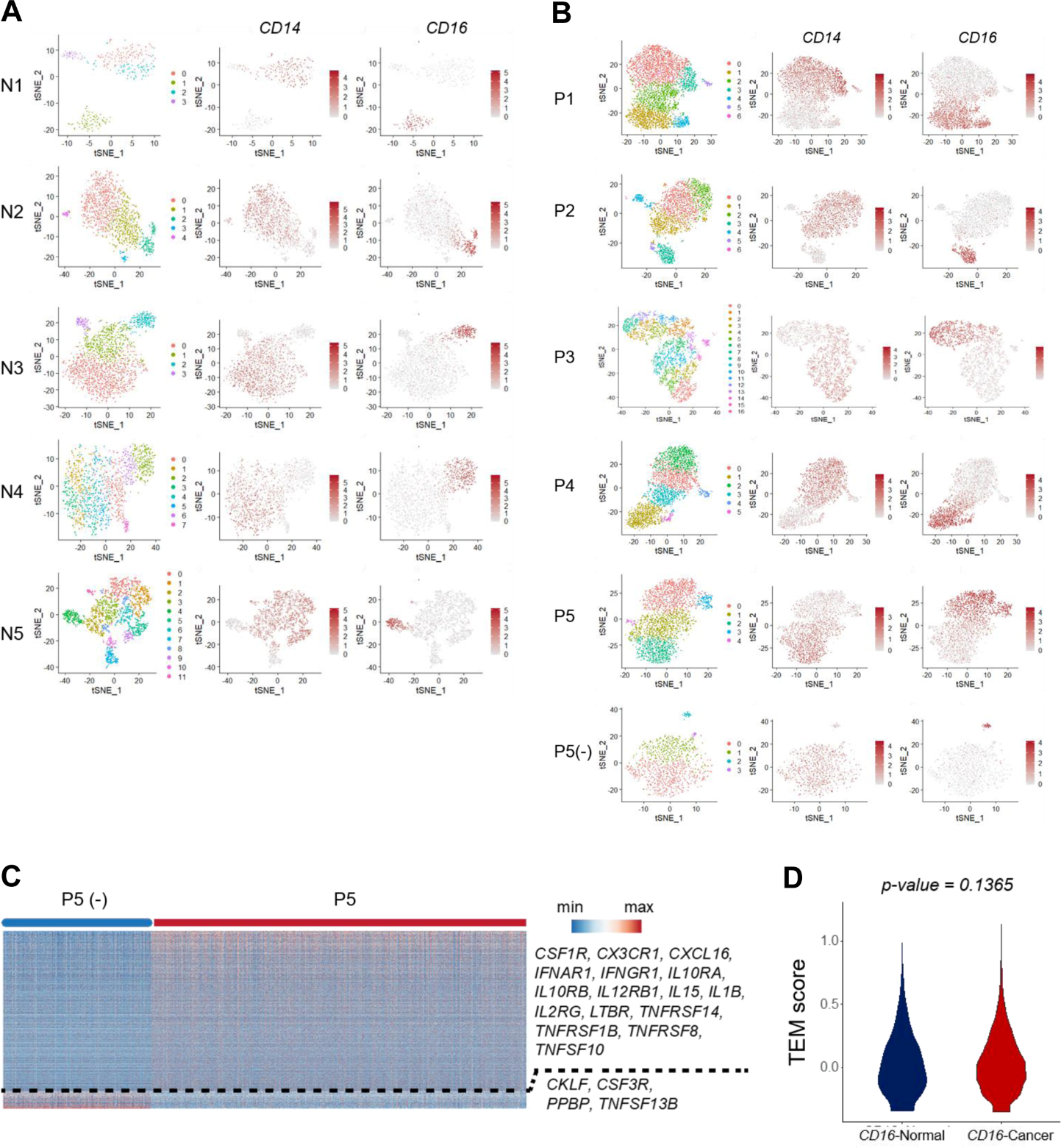


**Figure S4. Characterization of PBMC subtypes within the monocyte/macrophage compartment in PDAC patients.** (**A** and **B**) t-distributed stochastic neighbor embedding (t-SNE) plots of each sample colored by cell clusters (left), the expression levels of CD14 (middle), and CD16 (right) in normal subjects **(A)** and PDAC patients **(B)**. (**C**) Heatmap showing the relative expression (Z-score) of differentially expressed genes (adjusted P-value < 0.05) between IL10R2- (P5(-)) and IL10R2+ (P5) PBMCs from the same PDAC patient. (**D**) Violin plot showing the TEM signature score of CD16+ monocytes/macrophages between healthy donors and PDAC patients.


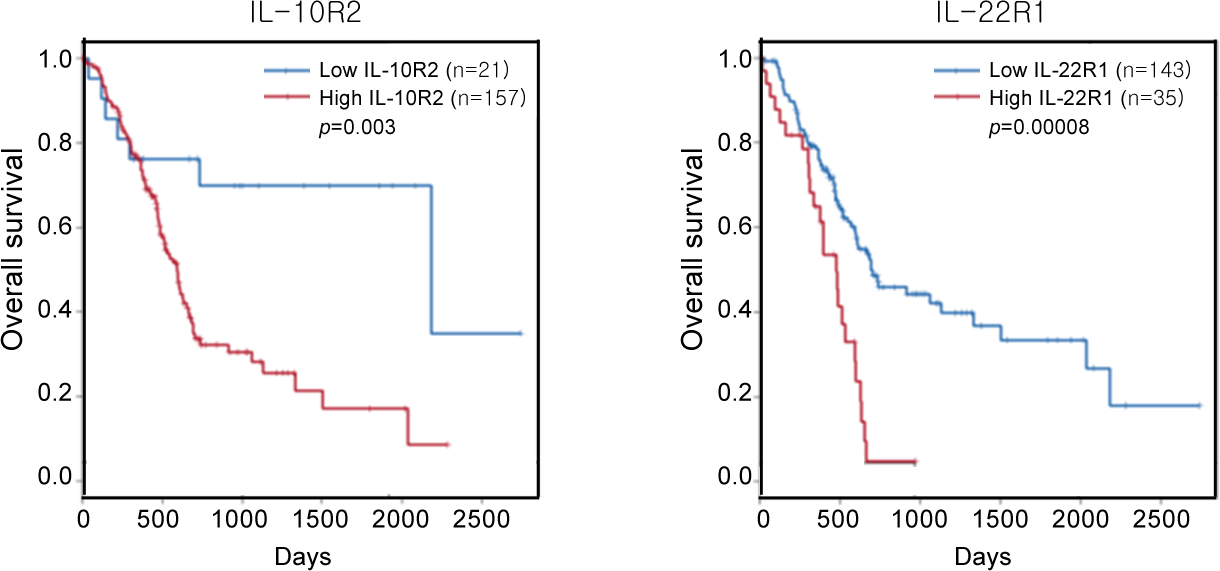


**Figure S5. Kaplan-Meier survival curves of PDAC patients with either high or low expression of IL10R2 or IL-22R1.** Kaplan-Meier survival curves using The Cancer Genome Atlas Program (TCGA) data revealed that there were worse overall survival rates for PDAC patients with high IL-10R2 expression compared to patients with low IL-10R2 expression (*p*=0.003). PDAC patients with high IL-22R1 expression had lower survival rates than patients with low IL-22R1 expression (*p*= 0.00008).
